# A Narrative Review Regarding Implication of Ovarian Endometriomas in Infertility

**DOI:** 10.3390/life15020161

**Published:** 2025-01-23

**Authors:** Constantin-Cristian Văduva, Laurențiu Dîră, Lidia Boldeanu, Mircea-Sebastian Șerbănescu, Andreea Carp-Velișcu

**Affiliations:** 1 Department of Obstetrics and Gynecology, University of Medicine and Pharmacy, Filantropia Clinical Municipal Hospital Craiova, 200143 Craiova, Romania; cristian.vaduva@umfcv.ro (C.-C.V.); laurentiu.dira@umfcv.ro (L.D.); 2 Department of Obstetrics, Gynecology and IVF, HitMed Medical Center, 200130 Craiova, Romania; 3 Department of Microbiology, University of Medicine and Pharmacy, County Clinical Emergency Hospital, 200642 Craiova, Romania; lidia.boldeanu@umfcv.ro; 4 Department of Medical Informatics and Biostatistics, University of Medicine and Pharmacy, 200349 Craiova, Romania; 5 Department of Pathology, Filantropia Clinical Municipal Hospital Craiova, 200143 Craiova, Romania; 6 Department of Obstetrics, Gynecology and IVF, “Carol Davila” Bucharest Medical University, Prof. Dr. “Panait Sarbu” Clinical Hospital, 060251 Bucharest, Romania; andreea_veliscu@yahoo.com

**Keywords:** ovarian endometriomas (OEs), infertility, endometriosis, ovarian sclerotherapy, in vitro fertilization (IVF)

## Abstract

Endometriosis is a multifaceted gynecological disorder defined by endometrium-like tissue outside the uterine cavity. It is mainly localized in the pelvis and creates a local inflammatory environment responsible for its manifestations and complications. In 30–50% of cases, endometriosis is associated with infertility. In 17–44% of cases, the ovaries are affected in the form of ovarian endometriomas (OEs). The symptoms of OEs are not very pronounced. The development is slow. Diagnosis is difficult because OEs resemble cystic ovarian pathology, which is so diverse. The actual diagnosis is possible through direct visualization or laparoscopy. Surgical treatment by cystectomy is common for OEs. Recently, other therapeutic modalities have emerged that have less impact on ovarian reserves and pregnancy rates. In this context, the review attempts to shed light on the best diagnostic and treatment methods for an insidious pathology with a major impact on fertility.

## 1. Introduction

Endometriosis is a systemic disease dependent on estrogen, characterized by pelvic pain and the impact on infertility, in which tissue resembling the inner lining of the uterus spreads outside the uterus [1]. This leads to an inflammatory condition due to endometrial-like glands and stroma in an abnormal location. The fallopian tubes, ovaries, and tissue lining the pelvis are often affected.

Endometriosis occurs most frequently in the pelvic region, but there are deep-infiltrating endometriosis, superficial implants, and solid nodules inside and outside the pelvis [2,3]. Implants can also occur throughout the abdomen, including the colon, in previous surgical wounds, and, in rare instances, in remote areas of the body, such as the cerebellum [4].

Typically, three different clinical manifestations are superficial endometrial implants in the peritoneum, OEs, and endometriotic nodules [5]. OEs consist of endometrium-like tissue in the form of ovarian cysts. They may be either invagination cysts or true cysts, with the cyst wall also containing endometrium-like tissue. These tumors are usually known as ‘chocolate cysts’ because of the viscous dark brown fluid they contain [6,7].

OEs signify a more advanced disease state in individuals with endometriosis and may result in complications, including diminished ovarian reserves [8,9].

Endometriosis is associated with an increased risk of infertility. Around 30–50% of people with endometriosis can become infertile. The probability of monthly conception in people without endometriosis is around 10–20%, while the probability in people with surgically confirmed endometriosis is around 1–10% [10]. In a population affected by subfertility, 17% have OEs [3,11].

This review aims to systematize the current knowledge on ovarian endometriomas (OEs), with a focus on etiology, diagnosis, and treatment for fertility preservation.

## 2. Epidemiology

Endometriosis mainly affects women of childbearing age. The estimated prevalence of endometriosis during a woman’s reproductive years is between 1 and 4%. OEs occur in 17–44% of women with endometriosis [12,13]. Bilateral OEs occur in 28% of these women [3].

The incidence of endometriosis is six times higher among first-degree relatives of women with severe endometriosis [14].

There are insufficient data to identify clear risk factors exclusively for OEs. Nonetheless, established general risk factors exist for the onset of endometriosis. Factors include nulliparity, early menarche, late menopause, short menstrual cycles, heavy menstrual bleeding, Müllerian anomalies, low body mass index, height exceeding 170 cm, high intake of trans-unsaturated fats, and in utero exposure to diethylstilbestrol [15,16,17]. A recent study found that the familial influence on the prevalence of endometriosis is negligible [18].

In addition to the risk factors associated with endometriosis, the disease carries other risks for patients, including infertility, persistent pelvic discomfort, dyspareunia, and dysmenorrhea.

## 3. Etiology

The many mechanisms of endometriosis development can explain the phenotypic heterogeneity observed in OEs.

Several theories have been described with supporting evidence.

1. Retrograde menstruation is the most widely accepted explanation [19]. Sampson hypothesizes that viable cells from the peritoneal fluid derived from retrograde menstruation can engraft, proliferate, and invade the peritoneal cavity [20]. Cells released from the endometrium during menstruation include epithelial cells, stromal fibroblasts/decidual cells, vascular cells, and various immune cells [21,22].

These cells can adhere to the surfaces of the pelvic organs. There, they can proliferate, thicken, and bleed with each menstrual cycle. Surprisingly, however, during menstruation, these cells do not adhere to the vaginal walls, which consist of stratified squamous, nonkeratinized epithelium and a high-pH vaginal environment. The prevalence of endometriosis in patients increases with the number of ovulatory menstrual cycles and with increasing life expectancy. Contradictions arise from the fact that partial retrograde menstruation is observed in most patients, although only 10% develop endometriosis [19].

2. Embryonic Müllerian remnants. This concept states that residual embryonic Müllerian migration cells retain the ability to grow into endometriotic lesions under the influence of estrogen, beginning around puberty or possibly in response to estrogen mimetics [12]. Epidemiologic studies indicate a twofold increased risk of endometriosis in women exposed to diethylstilbestrol in utero [23].
3. Genetic and epigenetic theory. Changes in the genetic regulatory networks have been found in the stromal cells of the endometrium [24]. Epigenetic alterations found in endometriosis include genomic DNA methylation [25]. In addition, aberrant DNA methylation in endometriosis induces the expression of many genes, including homeobox A10 (HOXA10), an estrogen beta receptor gene, a progesterone receptor, aromatase, HOXC6, and ALDH1A2 [26]. HOXA-10 is an important regulator of two essential processes during implantation: stromal cell proliferation and local immunosuppression [27].
4. Hematogenous and lymphatic spread. Another idea is that the menstrual tissue diffuses from the uterine cavity to distant areas via lymphatic vessels and veins, which explains the presence of implants outside the pelvis [28]. The blood contains endometrial cells derived from the endometrium. Microvascular studies revealed lymphatic movements from the uterine body to the ovary, indicating a possible involvement of the lymphatic system in the etiology of OEs [29].
5. Celomic metaplasia. Ferguson hypothesized that celomic metaplasia may play a role in the etiology of endometriosis. It is based on the hypothesis that the peritoneum harbors undifferentiated cells that can differentiate into endometriosis cells [5].
6. Neuroangiogenesis. Recent studies suggest that ectopic endometriotic implants attract a distinct neuronal and vascular supply via neuroangiogenesis. Endometriotic implants are complex multicellular structures characterized by vascularization that include the development of new blood vessels. It is postulated that the developing nerve fibers in the endometriotic implants influence dorsal root neurons in the central nervous system, thereby enhancing patients’ perception of pain [29,30].

Several chemokines have been shown to be elevated in the peritoneal fluid of women diagnosed with endometriosis. They may play a role in macrophage activation, inflammatory responses, adhesion of endometriotic tissue in the peritoneal cavity, and increased angiogenesis in the progression of endometriosis [31].

7. Stem cell theory. Progenitor cells within the endometrium and multipotent cells from the osseous sinus contribute to the composition of the eutopic endometrium [32].
8. Induction theory. Experts believe that hormones or immunologic factors may promote the transformation of peritoneal cells lining the internal abdominal cavity into cells resembling those of the endometrium [33].

In the typical endometrium, the expression of receptors for estrogens, androgens, progestins, and glucocorticoids is both cell-specific and dependent on the phase of the menstrual cycle. Estradiol promotes the synthesis of prostaglandin E2, which, in turn, increases aromatase activity. The expression of aromatase, a crucial enzyme in estrogen production, was detected in lesion-derived stromal cells more than 20 years ago [23].

These findings confirm the ability of endometriotic lesions to synthesize estradiol and validate treatments aimed at promoting a hypoestrogenic peritoneal microenvironment [19,34,35].

## 4. Infertility Related to OEs

The pathogenesis of OEs mirrors that of endometriosis. In endometriosis, the surrounding pelvic structures stick together, leading to the fusion of the fallopian tubes, ovaries, and intestines. Infertility due to OEs is characterized by deformation of the tubo-ovarian structure, impaired tubal motility, morphological distortion of the fallopian tubes, obstruction of oocyte retrieval, and peritubal adhesions.

Additional factors contributing to infertility in endometriosis include ovulatory dysfunction, abnormal folliculogenesis, anovulation, unruptured follicle syndrome, implantation failure due to endometrial dysfunction, peritoneal factors, local prostaglandin production, impaired coital function due to dyspareunia, and sperm inactivation due to macrophage phagocytosis [18]. The presence of OEs creates a pronounced prooxidant environment that negatively affects nearby ovarian follicles and, consequently, ovarian reserves [36].

The mechanism of this disease process involves the hormonal response of ectopic endometrial tissue. This tissue reacts to the cyclical hormonal fluctuations of a woman’s menstrual cycle similarly to the intrauterine endometrium. It will exhibit proliferative and secretory characteristics and undergo sloughing, like its behavior within the uterus. These oscillations result in differing quantities of cytokines and prostaglandin molecules.

Cytokines and prostaglandins serve as signaling chemicals that initiate an inflammatory response, hence producing inflammation at the site of the endometriotic implantation. This inflammatory reaction establishes the basis for new vascularization and the creation of fibrous tissue. This snowball effect subsequently generates adhesions and discomfort typically linked to this pathological process. These difficulties also result in the primary consequences of this condition, including infertility and chronic pelvic pain.

A significant relation has been demonstrated between OEs and pelvic deep-infiltrating endometriosis [2,37]. Furthermore, it is believed that dysregulation of the biomarkers involved in endometrial receptivity leads to endometrial progesterone resistance, increased cell proliferation, and decreased cell apoptosis, all of which ultimately contribute to subfertility [38].

Molecular, histological, and morphological findings suggest that OEs impair ovarian functions. The mechanism by which the OEs diminish the quantity of functional tissue—whether through a space-occupying action causing mechanical stretching damage or via a direct toxic effect—remains uncertain [32].

The presence of OEs leads to ovarian hypoperfusion and ischemia. The impairment of ovarian vascularity leads to a reduced gonadotropin response and, thus, to a decrease in follicular growth. One reason for the inadequate development of the follicles is seen in the fact that the mechanical pressure prevents the follicles from reaching a diameter of 18 mm [39].

**Figure 1:**
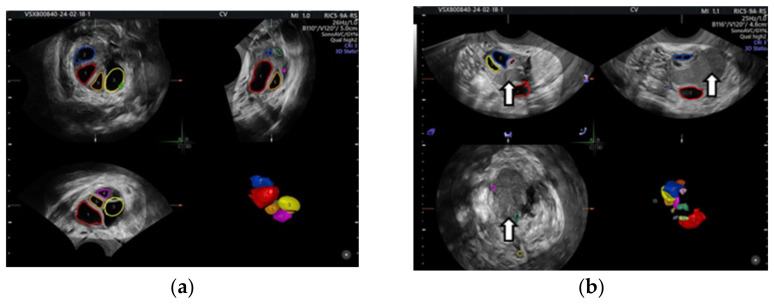
Color-coded stimulated follicles in the IVF cycle: (**a**) right ovary with normally growing follicles, (**b**) in the left ovary the arrows show an ovarian endometrioma compressing the follicles—vaginal 3D ultrasound with SonoAVC software (SonoAVC, automatic volume calculation: GE Medical Systems).

Even in the IVF cycle, the follicles stimulated with gonadotropin cannot grow sufficiently to be punctured. In the following figure, we show the case of a patient whom we stimulated for IVF but who was unable to get follicles to develop due to OE compression (Figure 1).

In addition, the presence of OEs made egg retrieval difficult and, in certain cases, made the ovary inaccessible. The ectopic endometrial tissue interferes with the normal intraovarian processes of follicular and oocyte maturation, thereby compromising the quality of the retrieved eggs. This does not appear to affect their ability to fertilize and divide but results in functionally impaired embryos that have difficulty implanting [40].

It is hypothesized that the fluid in the OEs has a high concentration of iron, which triggers cytotoxic oxidative stress that impairs the number of ovarian follicles and leads to their reduction in size [41].

The discharge of toxic cyst contents into the surrounding ovarian parenchyma may result in oxidative stress, fibrosis, the depletion of cortical stroma, smooth muscle cell metaplasia, compromised vascularization, and, in later stages, diminished follicular maturation and atresia in early follicles [42].

Recent studies have shown that the number of primordial follicles in human ovaries decreases with OEs. Luteinized granulosa cells from women with endometriosis exhibit increased apoptosis; granulosa cells from women with endometriosis exhibit a decreased expression of P450 aromatase and show altered progesterone release, possibly impairing proper oocyte maturation [43].

In addition, cryopreserved human oocytes subjected to endometriotic fluid from patients with advanced disease stages exhibited excessive cellular fragmentation in embryos, potentially resulting in compromised embryo development through the induction of apoptosis in adjacent blastomeres or by modifying blastomere division [44,45].

Peritoneal-fluid-derived macrophages can release proteinases that exert a deleterious effect on ovarian tissue and lead to a reduction in ovarian reserve. The main pathophysiological processes are inflammation, fibrosis, adhesions, and surgical sequelae. Anatomical distortions and mechanical variables may hinder oocyte release from the ovary, obstruct tubal ovum pickup or transport, and/or impede sperm transfer into the fallopian tube [46].

## 5. Symptomatology

The symptoms of OEs must be seen in the context of endometriosis.

Endometriosis can be asymptomatic or symptomatic. Typical manifestations of endometriosis may include menorrhagia, dysmenorrhea, dyspareunia, adenomyosis, gastrointestinal symptoms during menstruation, urinary symptoms during menstruation, increased pelvic mass due to massive OEs, and infertility [2,47,48].

Pain correlates with the extent of tissue infiltration as it is thought to be related to the degree of peritoneal inflammation rather than the number of implants [49].

One of the hypotheses is that painful symptoms in a woman with an OE may be caused by associated deep-infiltrating lesions of endometriosis (DIE). Associations between OEs and DIE are commonly found and can involve two severe forms: intestinal endometriosis and ureteral endometriosis. A histological study of adhesions in women with endometriosis has shown that periovarian adhesions contain endometrial and inflammatory cells that can cause painful symptoms. The evidence for a relation between OEs and painful symptoms is poor [50].

OEs may be scarcely neurotrophic and consequently not significantly related to pain [51]. In fact, scholars did not find nerve fibers in OEs [52]. Pelvic adhesions are probably more important than the OEs’ diameter, which is not correlated with the pain degree [2]. Upon the rupture of an ovarian endometrioma, the viscous endometrial fluid may disseminate throughout the abdominal cavity, resulting in considerable pain and inflammation. These individuals frequently exhibit an acute surgical abdomen [53].

Special attention should be paid in cases with localized adnexal pain to exclude cases of ovarian cancer. Numerous recorded instances exist of OEs occurring within abdominal surgical incision scars. Endometrial implants have been recorded in the lung parenchyma and the brain. Therefore, endometriosis should be considered when a patient reports cyclic discomfort during menstruation, regardless of where the pain occurs [5,54].

Patients with OEs associated with endometriosis have a more severe disease state and generally suffer more from this condition than those with stage one or two of endometriosis [2,37,38].

## 6. Diagnosis

The diagnosis of endometriosis often begins with the appearance of symptoms. It is based on the patient’s symptoms, physical examination and ultrasound (US) findings, magnetic resonance imaging (MRI), and blood tests. A normal result in these procedures does not rule out the disease.

### 6.1. Examination of the Pelvis

#### 6.1.1. Physical Examination

A thorough medical history and examination should form the basis of the examination. These are important in addition to the clinical symptoms of infertility described above.

A vaginal examination reveals cystic ovarian tumors, which may be associated with endometriosis, deposits at the vaginal level, and in the rectovaginal space, which is infiltrated. OEs may be palpable on bimanual examination if they are large enough. General pelvic discomfort or localized tenderness in the affected region is often observed. This may also be influenced by the time of the examination of the patient’s menstrual cycle. The patient typically has increased discomfort if the examination occurs shortly before the commencement of her menses. Additional potential findings during the bimanual examination may reveal a fixed or retroverted uterus, indicating scarring attributable to endometriosis. The uterosacral ligaments are hypertrophied, tender, and may be nodular [55,56,57].

Patients presenting after the rupture of OEs may show signs of an acute surgical abdomen on examination [52]. A combination of a physical examination and vaginal ultrasound is preferable [58].

#### 6.1.2. Laboratory Tests

This may include a complete blood count, cancer antigen (CA-125), chemokine receptor (CCR1), urinalysis, and tests for sexually transmitted infections. CA-125 is often elevated in women with endometriosis; however, its specificity for the disease is limited [23]. In situ hybridization has demonstrated a doubling of CCR1 microRNA (miRNA) transcripts from peritoneal cells in endometriosis [59]. During the last decade, new diagnostic tools have been investigated to detect this debilitating disorder as early as possible [60,61,62]. Among these, miRNA analysis uses a saliva-based diagnostic miRNA signature for endometriosis in the diagnosis care pathways after an external validation to confirm these results [63]. An elevated white blood cell count would increase the suspicion of an infectious etiology for the patient’s pelvic pain. Hemoglobin levels can provide insight into the extent of blood loss, as these patients often suffer from menorrhagia and may, therefore, be anemic. Urinalysis is also essential to exclude urinary tract infections, as are tests for sexually transmitted diseases, including cervical cultures.

Histologic evidence is usually obtained by detecting extrauterine endometrial cells during laparoscopy.

#### 6.1.3. Imaging

Originally, the US alone was not able to differentiate OEs from conditions such as tubo-ovarian abscesses, ruptured ectopic pregnancies, or other ovarian cysts and tumors [64,65]. The transvaginal US can accurately detect OEs with a sensitivity of 89% and a specificity of 91% and is considered the preferred imaging modality; however, smaller endometrial implants are not always detected [66].

**Figure 2:**
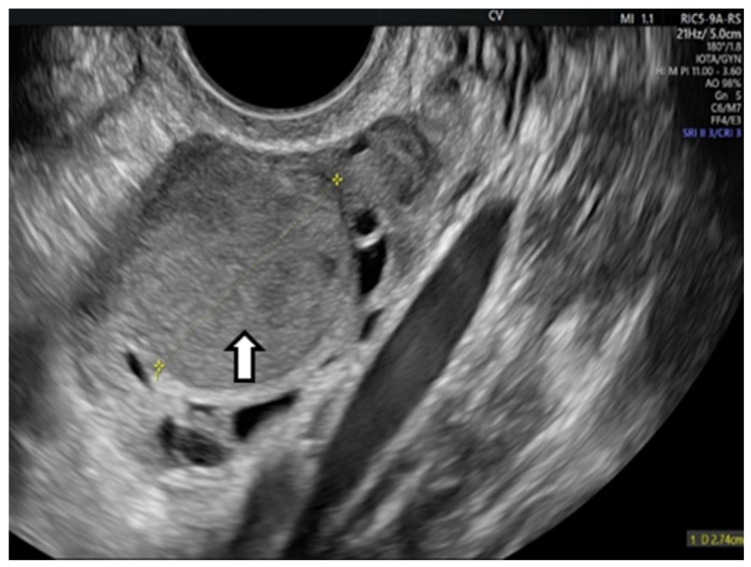
Vaginal ultrasound image of the ovary. The arrows show the ovarian endometrioma with ground-glass appearance.

OEs generally manifest as uncomplicated cysts. Nonetheless, they can also be classified as multilocular cysts or cystic solid lesions. The standard US presentation of these lesions has low-level homogeneous echoes, sometimes referred to as a ground-glass appearance. This aligns with previous hemorrhagic debris. These lesions generally lack vascularity when assessed by Doppler flow imaging. In the next figure, we present one of our cases [66,67] (Figure 2).

Also, the IOTA classification of simple descriptors, simple rules, and expert opinions performs well for classifying adnexal masses suspected to be OEs. The most common potentially malignant masses in these women were borderline ovarian tumors [68].

After the International Deep Endometriosis Analysis group, systemic endometriosis was diagnosed based on the presence of deeply infiltrative endometriotic nodules (for instance, uterosacral ligament, bowel, and bladder nodules), signs of pelvic adhesions (kissing ovaries or absent sliding of viscera, intestinal adhesions), or ovarian endometrioma or overt tubal disease [69,70,71,72,73].

Alternative imaging modalities include magnetic resonance imaging (MRI) and computed tomography (CT). MRI exhibits superior sensitivity in identifying pelvic masses compared to ultrasonography. Nevertheless, the expense of an MRI renders its advantages insufficient to justify the financial strain; hence, the US is utilized more frequently. Like US, an MRI is constrained in identifying widespread pelvic endometriosis and may primarily be advantageous for locating OEs. An MRI helps in the planning of surgery in certain individuals.

A CT scan, while exposing the patient to radiation, can reveal a pelvic mass, and the mass’s characteristics on the scan offer valuable insights into its kind. Consequently, a CT scan is not the optimal imaging technique for these patients.

A real-time dynamic assessment of pelvic organ motion is essential in all cases [64].

#### 6.1.4. Laparoscoy

**Figure 3:**
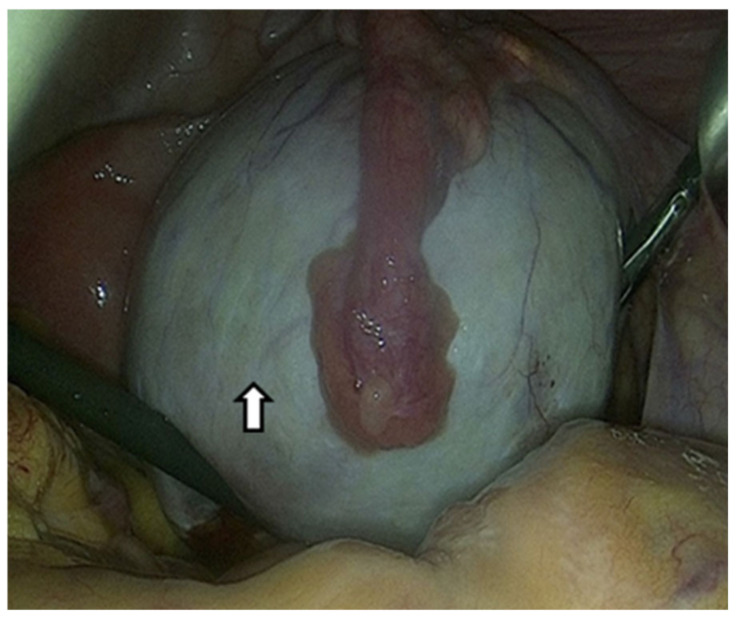
The arrow shows an ovarian endometrioma that has developed in the ovary and is centrally covered by the fallopian tube laparoscopic image. Suspected diagnosis based on the chocolate content and pathological confirmation by a tissue sample.

Due to the limited effectiveness of imaging techniques and laboratory tests in identifying endometriosis, the only method to confirm OEs is a surgical procedure that allows direct visualization and tissue sampling [74]. Laparoscopy can provide information on the location, extent, and size of OEs. The following figure shows an OE that we approached laparoscopically (Figure 3).

#### 6.1.5. Pathology

**Figure 4:**
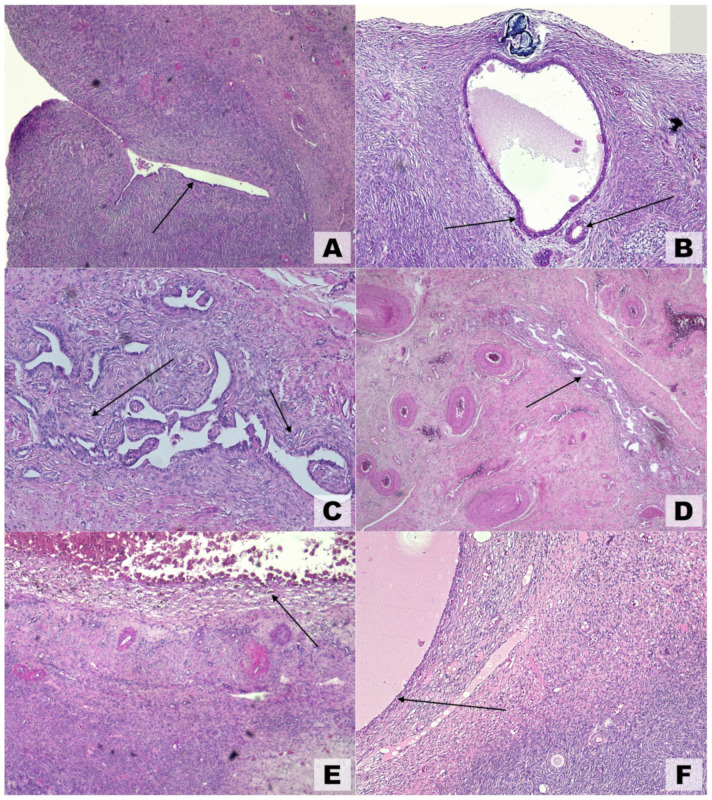
Microscopic findings in ovarian endometriosis using hematoxylin and eosin (H&E) staining. (**A**) Invagination of the ovarian cortex with colonization by endometrial-like epithelial cells (arrow), magnification 4×. (**B**) Early-stage endometriosis localized to the ovarian cortex; arrows highlight endometrial-like glands, magnification 10×. (**C**) Advanced cortical endometriosis characterized by stromal alterations that followed glandular changes (arrows), magnification 5×. (**D**) Medullary endometriosis (arrow) involving deeper ovarian layers, magnification 4×. (**E**) Hemorrhagic ovarian endometrioma displaying hemosiderin-laden macrophages (arrow) and degenerating blood components, magnification 4×. (**F**) Inclusion cyst shown for comparison, lacking endometrial-like epithelial lining (arrow), magnification 4×.

Because they are generated by extraovarian parenchyma, OEs have been referred to as pseudocysts and exhibit unique anatomical features that are not shared by other benign ovarian cysts. These results clarify why surgical enucleation entails ovarian reserve reduction, follicle loss, and partial gonadal cortex removal [75]. Macroscopic and ultrasonographic findings are typically corroborated by microscopic pathological observations (Figure 4). Various theories have been proposed to explain the development of ovarian endometriosis. One such theory, illustrated in Figure 4A, suggests the colonization of an ovarian cortical invagination by endometrial-like cells [20]. Another theory, depicted in Figure 4B, proposes an internal (embryonal) origin for endometriosis, where the condition arises without evidence of external colonization.

However, they may also be accompanied by blue, red, white, or unpigmented endometriotic implantation lesions on the peritoneum. The presence of OEs, significant adhesions, and peritoneal deformities indicate more serious endometriosis. This aspect is obvious when we look at the following table that presents the revised American Society of Reproductive Medicine (ASRM) staging system (Table 1) [38].

The visualized lesions might undergo biopsy and subsequent pathological evaluation for endometrial glands and stroma [76]. Should the patient encounter infertility concerns, chromotubation may be performed concurrently to evaluate tubal patency.

### 6.2. Differential Diagnosis

When assessing individuals with suspected OEs, it is crucial to investigate all potentially associated diseases [15]. Patients often present with non-specific pelvic symptoms. Therefore, other causes of pelvic pain should also be considered in the differential diagnosis. These include ectopic pregnancy, pelvic inflammatory disease, appendicitis, diverticulitis, ovarian torsion, urinary tract infection, ovarian cysts, and sexually transmitted infections [50,77].

As mentioned in the assessment section, OEs show a distinct ground-glass appearance in the US image. These observations are similarly evident in hemorrhagic cysts, and frequently, the differentiation between the two is not established until surgical intervention [78].

When a patient presents with symptoms of an acute surgical abdomen and a ruptured OE is suspected, it is essential to consider ruptured ectopic pregnancy and ovarian torsion as primary differential diagnoses [50,79]. These are all surgical emergencies that require immediate transfer to the operating room.

## 7. Complications

Complications can be divided into clinical conditions due to the OEs’ size, the endometriotic tissue penetration, o adhesion, perforation, or a malignant change.

### 7.1. Risk to Fertility

According to previous studies, endometriosis impairs the function of the ovaries by damaging the ovarian tissue. To prevent spontaneous ovulation, endometriosis can impair physiological reproductive processes [80]. Reduced ovarian reserves may be caused by endometriosis and associated structural tissue changes in the ovarian cortex [81].

According to reports, women with endometriosis have a monthly fertility rate of 2 to 10%, which is lower than the rate of 15 to 20% in healthy women. Furthermore, the more severe the disease, the lower the fertility rate [40].

### 7.2. Risk of Infection or Contamination During IVF Cycles

Several substances found in OEs are thought to be harmful to eggs. Smaller studies suggest that such contamination may decrease the fertilization or pregnancy rate, so it is necessary to avoid the accidental puncture of the OEs and to wash the oocyte immediately if contamination is detected [82].

Infection in OEs can result from an accidental puncture during oocyte retrieval or iatrogenic damage after oocyte retrieval. Although prophylactic antibiotics cannot completely prevent these infections, they are important to reduce the risk of infection when an endometrial puncture is suspected [40].

### 7.3. Risk of Egg Retrieval

Ovaries are sometimes displaced to regions that are difficult to access (e.g., behind the uterus) and do not glide due to adhesions; the presence of OEs is thought to exacerbate difficulties with egg retrieval. This can lead to certain problems. First and foremost, there is a risk of rupture of the OEs. This event can lead to chemical peritonitis and unexpected severe pain [83]. Damage to other organs, particularly the bowel, is a possible second concern.

### 7.4. Risk for Pregnancy

Delayed implantation can lead to placenta previa or placental insufficiency, which can cause intrauterine growth restriction, preeclampsia, and obstetric hemorrhage. Recurrent pregnancy loss and spontaneous abortions can also be the result of endometriosis-related implantation that occurs outside the typical implantation window. According to Leone Roberti et al., premature decidual senescence can lead to premature birth [84]. Despite conflicting results from epidemiologic research, there is currently no evidence that preventive surgery halts the deleterious effects of endometriosis on pregnancy outcomes [81].

### 7.5. Risk of Disease Development During IVF Cycles

As endometriosis is an estrogen-dependent disease, the question arises as to how regulated ovarian stimulation for ART may affect the progression or recurrence of the disease. The natural history of endometriotic lesions did not appear to be affected by gonadotropin treatment, and the patients who received ART had a cumulative recurrence rate comparable to the control group [85].

Pregnancy in mothers who already have endometriosis has become an important issue due to the effectiveness of ART. According to Ueda’s results, in these situations, 52% of patients experienced a decrease in OEs’ size, 28% had no change, and 20% had an increase [86]. The OEs showed signs of rupture, abscesses, and decidualization. According to other studies, endometriosis during pregnancy can be studied with caution as most lesions do not shrink or change in size [87].

### 7.6. Other Complications

Risk of adhesion—OEs can lead to local problems because endometrial tissue adheres and penetrates other organs, e.g., injury to the rectum, bladder, or fallopian tubes.

Risk of perforation—in more severe cases, these OES can perforate and cause symptoms of an acute surgical abdomen.

Risk of torsion—if the OEs measure 6 cm or larger, the patient has an elevated risk of ovarian torsion, a surgical emergency that may result in the loss of the ovary [88].

Risk of malignant change—OEs present a minor risk of malignancy, particularly epithelial ovarian malignancies [89]. Nonetheless, other research suggests that women with endometriosis have a fourfold increased risk of certain forms of ovarian cancer: clear cell and endometrioid ovarian cancer [90]. In a specific study from Finland, an increased risk was only found in women with OEs [91]. Gene activation of KRAS and PI3K/AKT may be linked to ovarian cancer and a history of ovarian mucinous adenomas. Moreover, genes like PTEN and ARID1A have been associated with the etiology of endometrial cancer [92]. Nevertheless, these lesions by themselves are not considered premalignant lesions, and no staging evaluation or screening is necessary.

## 8. Treatment

### 8.1. Surgical Treatment

If a patient’s endometriosis is so advanced that OEs have developed, surgery is often preferred, especially laparoscopic or robotic procedures.

If a patient’s discomfort is intense and they do not wish to preserve future fertility, some patients opt for a total hysterectomy with bilateral salpingo-ophorectomy as a more conclusive treatment.

For conservative treatment, there are other surgical procedures: cystectomy, enucleation, partial oophorectomy, ablation by laser or by plasma energy or electrocoagulation, and laparoscopic fulguration. The disadvantages include a high recurrence rate and a decrease in ovarian reserves [3].

The European Society of Human Reproduction and Embryology (ESHRE) guideline recommends considering surgery prior to assisted reproductive technology only in women with ovarian masses larger than 3 cm and only to relieve endometriosis-related symptoms or improve follicular accessibility [93].

Ovarian cystectomy is the favored method for recurrence and the rate of spontaneous pregnancy post-surgery [3]. The primary concern of surgical OE excision, particularly in women facing infertility and contemplating in vitro fertilization (IVF), is its impact on ovarian reserves. Cystectomy often leads to ovarian impairment and reduced ovarian reserves. Three months after ovarian cystectomy, serum levels of the anti-Müllerian hormone (AMH) decreased by 36% and 49% in patients with unilateral and bilateral ovarian masses, respectively, due to the surgical removal of endometrial and normal ovarian tissue [8,9,94]. In addition, the remaining normal ovarian tissue is usually coagulated to control bleeding, which further reduces the ovarian reserve [95]. In older women and women with larger ovarian masses, bilateral lesions, and advanced stages of disease, the ovarian reserve may be more severely reduced. Therefore, cystectomy must be chosen with extreme caution in women who wish to have children or are infertile. To preserve the ovarian reserve during a cystectomy, hemostasis was introduced by suturing the ovaries or by using a hemostatic agent [96].

For extensive OEs, a three-stage protocol may be recommended, starting with a laparoscopic procedure to drain the cyst, followed by three months of treatment with gonadotropin-releasing hormone (GnRH) agonists and a second laparoscopy to ablate the reduced ovarian endometrioma [97].

Ablation is a technique for incising OEs to extract the internal fluid and ablate the endometrial lining, and it is seen as a superior alternative to cystectomy concerning ovarian reserves. Ablation may be executed with bipolar coagulation, laser vaporization, or plasma energy [98].

When surgically resecting OEs, it is crucial to remove the cyst wall and not just aspirate the cyst contents. This has been shown to reduce the recurrence rate. However, resection of OEs has been shown to increase the spontaneous conception rate in patients with reproductive problems [99].

The reduced AMH levels indicate that both ablation and cystectomy impair ovarian reserves. Recent studies have shown that the size of OEs correlates with reduced AMH levels after ablation or a cystectomy [100]. Consequently, for OEs of ≥5 cm, ablation may be preferable to a cystectomy to preserve serum AMH levels [101].

In addition, there is a documented incidence of 2 to 3% of patients experiencing ovarian failure after the removal of bilateral ovarian masses [102]. Given the limited data available, OEs are often treated expectantly in patients under the care of a fertility specialist. Exceptions are made for severe symptoms or complications during egg retrieval due to OEs [96].

Endometriosis may result in many adhesions and endometriotic implants. Occasionally, these lesions may affect the colon or the bladder. In advanced illnesses, intestinal blockages or ureteral involvement may occur. In such instances, general surgery or urology may need to participate in executing the requisite surgical interventions [103,104].

### 8.2. Medical Treatment

Although medical treatment can reduce the size of OEs, its main aim is to control the symptoms and slow the progression of the underlying disease.

#### 8.2.1. Suppression of Ovarian Function

Since steroidogenesis is disturbed in endometriosis, hormone suppression therapy is considered. Hormone treatment options include oral contraceptives, progestins, aromatase inhibitors, gonadotropin-releasing hormone (GnRH) agonists and antagonists, and androgens [105]. These medical treatments are primarily based on methods that inhibit ovarian function. They reduce the effect of estrogen on the endometriotic tissue and reduce the pain associated with endometriosis. However, the drugs administered for this purpose usually lead to contraception or subfertility, so these treatments are not helpful for the treatment of endometriosis-associated infertility [93]. Eight international guidelines recommend progestogens as the first-line medical treatment for endometriosis [106].

Hormone therapy as a neoadjuvant or adjuvant to surgical therapy is not effective in the treatment of infertility [107]. However, a recent Cochrane review found that drug suppression before surgery can prevent recurrence and increase pregnancy rates [107]. An effective adjuvant postoperative strategy to prevent or reduce the frequency and severity of recurrent dysmenorrhea and morphologic recurrence of endometriosis is long-term contraceptive use [108].

#### 8.2.2. Symptomatic Therapy

Non-steroidal anti-inflammatory drugs and analgesics are very effective against endometriosis-related pain. They are recommended as first-line medical therapies due to their easy accessibility as over-the-counter medications and low side-effect profile [50].

#### 8.2.3. Other Medical Therapies

According to an international survey, 49% of people with endometriosis have used cannabis to treat their symptoms [109]. Other medications have also been used, such as selective estrogen or progesterone receptor modulators, which act on estrogen or progesterone receptors [106]. In addition, palmitoylethanolamide has anti-inflammatory and analgesic properties [110].

### 8.3. Other Alternative Treatments

#### 8.3.1. Sclerotherapy Is a Non-Surgical Approach to the Treatment of OEs

The epithelial cells lining the walls of ovarian OEs play a crucial role in the cellular mechanisms of sclerotherapy. Sufficient interaction between the sclerosing agent and the cyst wall activates the coagulation cascade, producing inflammatory mediators, which then trigger fibrosis by the cells lining the endometrioma wall, eventually causing adhesion of the walls. The result is the destruction of pseudo-capsule cytoarchitecture [111].

Agents used in sclerotherapy include ethanol, methotrexate, interleukin-2, recombinant interleukins, metronidazole, tetracycline, leuprolide, and cefoperazone-sulbactam. The predominant method is transvaginal sclerotherapy with ethanol. The optimal ethanol concentration and quantity for the sclerotherapy of OEs have not yet been determined [112].

**Figure 5:**
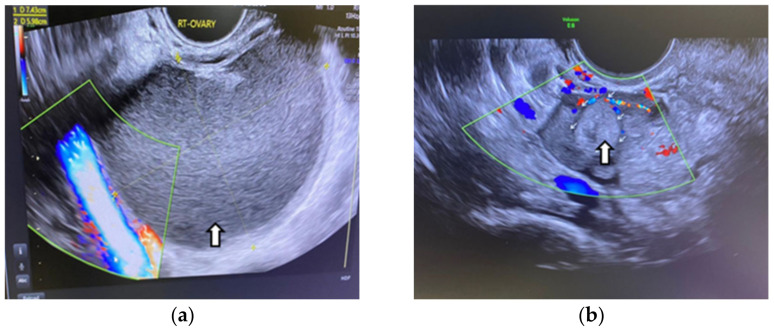
Vaginal ultrasound image of an ovarian endometrioma treated by sclerotherapy: (**a**) Arrow shows the original aspect of the endometrioma (**b**) Arrows show the endometrioma 6 months after ethanolic sclerotherapy. A considerable reduction in size can be seen.

In sclerotherapy, an OE is punctured directly transvaginally or percutaneously to extract the internal fluid, and then a sclerosing agent, like ethanol, is introduced into the cyst cavity and removed again after a certain period (“flushing”). A direct puncture can be performed with a long aspiration needle (16–17 gage) or a flexible catheter (catheter-guided sclerotherapy). US-guided aspiration and ethanol sclerotherapy is indicated for OEs with an average diameter of 4–8 cm, and the reduction in size at 6 months can be major (7–8 times), as we see in the next figure from our previous article [9] (Figure 5).

Although there is no evidence of malignancy or pelvic abscess, many gynecologists avoid puncturing OEs [12].

Sclerotherapy with ethanol is associated with a minimal recurrence rate of 7.4% at 6 months [9]. The recurrence rate correlates with the duration of instillation, as prolonged instillation is associated with an increase in cystic fibrosis. Cohen et al. found that ethanol and tetracycline can cause abdominal pain and increase the recurrence rate, while methotrexate can cause postoperative fever [111].

A retrospective review of recurrent OE cases found that the 1-to-10 min wash method had a non-significantly increased recurrence rate at one year compared to the retention method (32.1% vs. 13.3%) [112]. Consequently, it is uncertain whether the irrigation technique has a higher recurrence rate compared to the retention technique in the sclerotherapy of OEs [113].

Sclerotherapy for the treatment of ovarian masses up to 8 cm in size is a simple procedure that significantly reduces the likelihood of recurrence while preserving the ovarian reserve [114]. Laparoscopic cystectomy comes with the resources, costs, and risks associated with any surgical procedure. In addition, the ovarian reserve is significantly impaired, resulting in reduced fertility in patients who have undergone surgery. Sclerotherapy is suitable for individuals seeking IVF treatment [9,115].

#### 8.3.2. Drainage of Cysts

The simplest and least invasive technique for removing cysts is technical cyst drainage, in which a needle is inserted into the cyst wall to aspirate the contents. This procedure can be performed either transvaginal or laparoscopic under ultrasound guidance.

However, it has been observed that the recurrence rate of OE can be between 80% and 100% [116].

Assisted reproductive techniques (ART) are therapeutic modalities that correct the effects of OEs on fertility.

Controlled ovarian stimulation and intrauterine insemination (IUI) can be a useful alternative treatment for patients with mild-to-moderate endometriosis and infertility [117]. In women with severe endometriosis, such as OEs, IUI is generally not used due to impaired tubal function and concerns about pelvic adhesions. In these cases, IVF should be considered [118].

As mentioned above, OEs appear to reduce ovarian reserves, which may lead to a reduced ovarian response to IVF gonadotropin stimulation. However, other findings contradict this conclusion. According to a retrospective study of IVF treatment cycles, the size or amount of OEs did not correlate with the number of oocytes retrieved [119].

Although moderate-to-severe endometriosis appears to affect IVF outcomes, a systematic review comparing the ART outcomes of women with OEs without surgical treatment with those of women who underwent OE removal prior to IVF found similar clinical pregnancy rates, live birth rates, and the average number of eggs retrieved [120].

According to the ESHRE guidelines, surgical removal is not routinely recommended before ART is considered. When deciding whether to surgically remove endometriotic lesions, clinicians are advised to assess ovarian reserves. To determine the best treatment option, it is recommended to monitor AMH levels for at least three months after surgery [93].

If conception does not occur within six to twelve months after OE surgery, IVF is recommended. The various options for preserving fertility include the cryopreservation of embryos or eggs and the cryopreservation of ovarian tissue [121].

In summary, the treatment of OEs can be presented schematically, depending on the patient’s symptoms and her desire for future fertility (Table 2).

## 9. Prognosis

The general prognosis for people with endometriosis is positive. It is a non-threatening disease. Nevertheless, it is a persistent disease that can progress. Patients with OEs indicate a more severe disease state and, thus, may experience greater long-term repercussions from the condition. Although treatment can be temporarily effective for patients, the disease is unfortunately characterized by a high relapse rate. Fortunately, symptoms improve in most women during menopause, which is due to the absence of cyclical hormonal signals [3].

## 10. Conclusions

OEs are a frequently encountered pathology in gynecological practice. The presence of OEs reduces the number of ovarian follicles and creates a toxic environment with negative effects on the oocytes and the development of the embryos resulting from their fertilization. The diagnosis is difficult because the symptoms appear gradually, and the differential diagnosis with extensive cystic ovarian pathologies is controversial. Treatment depends on the clinical context and the patient’s desire to preserve her fertility. Treatment is mainly surgical, and the most important question for gynecologists is whether OEs should be removed or not. Of the available surgical methods, cystectomy appears to be advantageous in terms of the lower recurrence rate and the likelihood of spontaneous conception. Current therapeutic options have evolved, and recently, sclerotherapy using US-guided punctures has gained acceptance. Drug therapy can be used as an adjuvant therapy. ART is a viable alternative for the treatment of infertility associated with OEs when other procedures fail to deliver results.

## Figures and Tables

**Table 1:** Stages of endometriosis.

Stage	Classification	Description
I	Minimal	Few superficial implants
II	Mild	More implants, deeper involvement
III	Moderate	Many implants, small OEs, adhesions present
IV	Severe	Many deep implants, large OEs, many adhesions

**Table 2:** Surgical treatment of ovarian endometrioma.

Desire for natural conception	Indication: intact ovarian reserve, unilateral cyst, without surgical treatment Surgery—combined ablation and cystectomy
Reproductive surgery to improve IVF results (pain or other symptoms, male or tubal infertility, women who do not want to wait for natural conception after surgery)	Indication: large cysts Surgery—three-stage techniques or aspiration with sclerotherapy followed by IVF with GnRH agonist’s long protocol
Expectant management and IVF (no pain symptoms, male or tubal infertility)	Indications: low AMH, small cysts, bilateral OEs, previous surgical treatment IVF after 6 months of treatment with GnRH agonists

## Data Availability

Not applicable.
